# Visualizing lens epithelial cell proliferation in whole lenses

**Published:** 2010-07-09

**Authors:** Luke A. Wiley, Ying-Bo Shui, David C. Beebe

**Affiliations:** 1 Department of Ophthalmology and Visual Sciences, Washington University, Saint Louis, MO; 2 Department of Cell Biology and Physiology, Washington University, Saint Louis, MO

## Abstract

**Purpose:**

To develop a means to image cells in S-phase of the cell cycle while preserving the anatomic relationships within the lens.

**Methods:**

Mice were injected with the thymidine analog, EdU. Whole lenses were removed, fixed and permeabilized. Cells that had incorporated EdU into their DNA were chemically labeled using fluorescent azides and “click” chemistry. Double labeling was performed with antibodies to other antigens, like phospho-histoneH3, a marker of mitotic cells. The position of labeled cells and lens anatomy was viewed using a simple device to position and flatten the lens.

**Results:**

The nuclei of cells in S-phase of the cell cycle were intensely stained without the use of antibodies. Stained cells were readily localized with reference anatomic landmarks, like the transition zone. Whole lenses could be assayed by rotating the lens on the microscope stage. Double-labeling permitted the co-localization of markers in cycling cells.

**Conclusions:**

EdU labeling of whole lenses provides a simple, rapid and sensitive means to analyze lens epithelial cell proliferation in the anatomic context of the whole lens.

## Introduction

Quantification of cells in S-phase is useful for the quantification of cell proliferation and to provide insight into growth patterns of cells and tissues. Detection of cells in the process of DNA synthesis involves incorporation of labeled DNA precursors into cellular DNA during replication. For years, this was accomplished using radiolabeled [^3^H]-thymidine, followed by sectioning and detection by autoradiography. Later, [^3^H]-thymidine was replaced by 5-bromo-2’-deoxyuridine (BrdU), in which detection is achieved using antibodies against BrdU-containing DNA [1,2]. Although useful, these methods have limitations, especially with respect to time, the need for dissection or sectioning of the tissue, and harsh treatment of the samples.

Recently, a more efficient means was developed to label S-phase cells using 5-ethynyl-2’-deoxyuridine (EdU) [3,4]. EdU is a thymidine analog in which the methyl group is replaced with a terminal alkyne group. This terminal alkyne can be conjugated to commercially-available, fluorescently-labeled azides, using copper-catalyzed “click” chemistry. This method does not require the DNA to be denatured, avoiding harsh acid treatment, and speeds the labeling process by avoiding the need for antibody staining and washing.

Here, we report the use of EdU to visualize and quantify S-phase cells in intact, adult lenses. This approach allows for detection, imaging, and quantification in one day, instead of the 2–4 days required for standard BrdU labeling. Furthermore, detection in whole lenses preserves the spatial relationships that are often distorted when lenses are sectioned or when lens explants are dissected for BrdU labeling. Assessment of lens cell cycle kinetics in vivo could lead to new insight into the control of lens growth during aging, which could be important, since epidemiologic studies showed that having a smaller or larger lens is a risk factor for the development of cortical or nuclear cataracts, respectively [5,6].

## Methods

### In vivo labeling of S-phase cells with EdU

**Figure 1:**
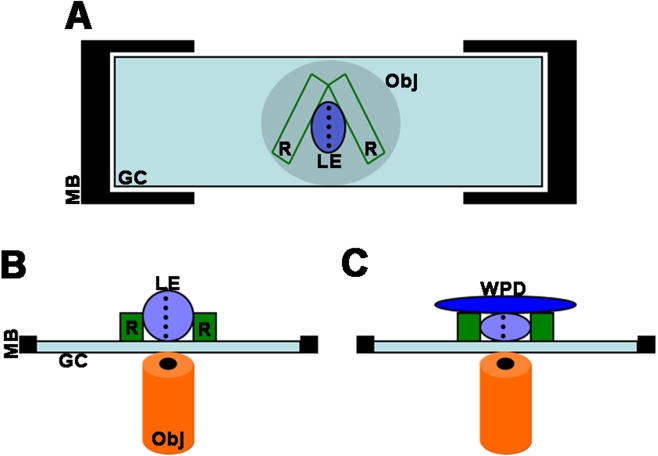
Apparatus used to image whole lenses. **A**: Overhead view of the apparatus. Two pieces of rubber (R) glued to a glass coverslip make a “wedged” well in which to hold and stabilize the lens (LE) on its equator (dashed line). The coverslip can then be held in place on the microscope stage and imaged from below by the microscope objective (Obj). Because the lens is held stationary in the rubber well, it can gently be rotated using forceps. In this way, the entire lens equator, including the transitional and germinative zones can be imaged. **B**: Side view; the lens is still in its normal, spherical shape. When rounded, the image plane is small. **C**: En face view of the set-up after gently applying a weighted Petri dish (WPD) on top of the lens. This flattens the fixed lens, allowing for capture of full image fields.

Mice were injected intraperitoneally with 5-ethynyl-2’-deoxyuridine (EdU) (Invitrogen, Carlsbad, CA) 1 h before death. One-month-old mice received 100 µg of EdU and 8-month-old mice were given 200 µg. Eyes were enucleated, whole lenses isolated, and any adherent ciliary epithelium removed from the lens by brief treatment with 3 mg/ml chymotrypsin (Sigma Aldrich, St. Louis, MO) in balanced salt solution (BSS). Removal of the ciliary epithelium is critical for visualizing the germinative zone near the lens equator. Lenses were fixed in 10% neutral-buffered formalin in 1× PBS at room temperature (RT) for at least 10 min. After rinsing, lenses were permeabilized in 0.5% Triton-X100 (Fisher Scientific, Pittsburgh, PA) in 1× PBS for 1 h. Lenses were then stained for EdU detection with AlexaFluor 488-azide using a Click-iT^™^ Kit for one hour according to manufacturer’s instructions (Invitrogen). Total nuclei were counterstained with DRAQ-5 (Biostatus Limited, Shepshed, Leicestershire, UK) for 30 min at RT in 1× PBS at a dilution of 1:2,000. Lenses were then rinsed in 1× PBS containing 0.03% sodium azide at 4 °C for 1 h before imaging. For visualization of the germinative zone, lenses were positioned on their equatorial surface in a homemade imaging apparatus (Figure 1). Lenses were placed on a glass coverslip (24×60–1.5; #22 266 882; Fisher Scientific) in between two pieces of adhesive rubber that served as an anchoring channel (200 µl Rubber CoverWells; #002PC200; Surgipath Medical Industries, Inc., Richmond, IL). One cover well was cut into quarters, with each quarter having the approximate dimensions of 2.5 cm long × 1 cm wide × 0.2 cm high. Two of the four pieces were then placed across the width of a glass coverslip at an approximate angle of 5°, creating a wedge-shaped well (Note to readers: these cover wells are available in various heights (thicknesses), which might be useful to account for the varying sizes (ages) of the lenses being investigated). Lenses were kept moist with a drop of 1× PBS and then gently compressed with a weighted Petri dish to flatten their equatorial surfaces, allowing for more efficient imaging of the lens equator. Lenses were viewed using a Zeiss 510 confocal microscope (Carl Zeiss Microimaging, Inc., Thornwood, NY,). Images were quantified using the NIH image public software, Image J64.

### Immunofluorescent antibody detection in whole lenses

Whole lenses that had incorporated EdU were isolated, fixed, and permeabilized as described above. Lenses were incubated in blocking buffer consisting of 5% normal goat serum, 0.5% Triton-X100, and 0.03% sodium azide with a fluorescent-labeled primary antibody or fluorescent phalloidin at 4 °C overnight with gentle agitation and then rinsed in 0.5% Tween-20 in 1× PBS for 2 h at room temperature before being stained for EdU, as described above. Primary antibodies/stains used in this study were AlexaFluor555-conjugated phospho-Histone H3 (Ser10) at a 1:500 dilution (Cell Signaling, Danvers, MA) and AlexaFluor488-conjugated phalloidin (for F-actin) at a 1:1,000 dilution (Molecular Probes, Eugene, OR).

### Statistical Analysis

Labeling indices were compared using Student’s unpaired *t*-test. Error bars are ±SEM.

## Results and Discussion

### Imaging intact lenses

Quantifying lens cell proliferation has usually been done on lens epithelial explants or lens sections. For adult lenses, explants provide more useful data, since the relatively low labeling index means that many sections will have no labeled cells. However, dissection of the lens epithelium to produce an explant is tedious, time consuming, and requires practice. This is especially true in older rodent lenses or human lenses in which the thick capsule hinders attachment to plastic tissue culture dishes. Isolation of the lens epithelium also causes mechanical damage, including loss of cells.

To address this problem, we developed ways to label whole lenses and a novel apparatus for imaging them (Figure 1). The imaging system is easy to assemble and affordable. Two pieces of rubber, cut into rectangles were glued to a standard glass coverslip and angled to make a V-shaped well (Figure 1A). In this way, lenses of various sizes can be used with the same holder. Lenses can easily be oriented on their equators by gently wedging them into the well. The coverslip is held on the microscope stage by an adjustable sample holder.

Looking at the apparatus from the side, the lens rests against the coverslip, allowing for visualization by the inverted microscope objective (Figure 1B). The curved surface of the lens permits the visualization of only a small number of cells in the focal plane. To address this, we applied a small, weighted Petri dish on top of the stationary lens. The rubber guides limited the amount of compression. This gentle pressure flattens the lens equator against the coverslip, permitting the visualization of a larger extent of the lens surface. It is also possible to use a confocal microscope to acquire a Z-stack of images from an uncompressed lens, from which a projection can be made. Combining both techniques permits the visualization of an even larger fraction of the lens surface.

Another advantage of this system is that, once the lens is stabilized on its equator, it can be rotated. Rotating the lens allows one to image the germinative zone around the circumference of the lens. Using lens explants, the area used for data collection varies greatly, depending upon success of the dissection. Thus, this system allows for quantification of lens epithelial proliferation in an entire lens.

### Labeling S-phase cells in whole lenses

To label S-phase cells in whole lenses, we injected mice with the thymidine analog, 5′-ethynyl-2’-deoxyuridine (EdU). After incorporation of the label into DNA, S-phase cells were detected by chemically coupling fluorescent dyes directly to the modified nucleosides, using a copper-catalyzed (“click”) reaction, without the need to denature the DNA, as is required for detecting BrdU-labeled DNA with antibodies.

**Figure 2:**
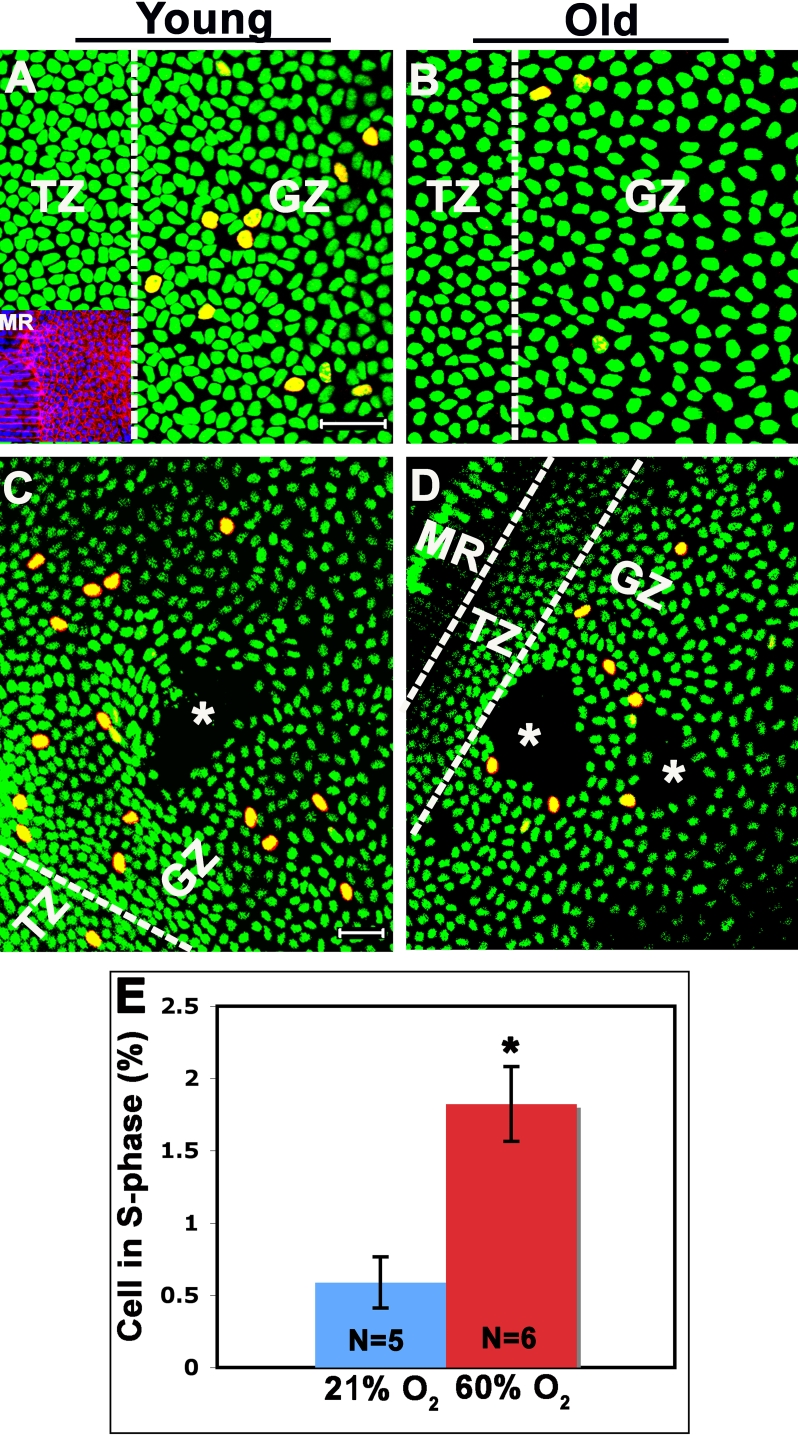
Comparison of whole lenses labeled with EdU and lens explants labeled with BrdU. **A**: EdU labels lens epithelial cells in S-phase in the germinative zone (GZ) of a 1-month-old lens. Lenses were placed on their equators and image fields were captured after moving one cells width anteriorly from the meridional rows (MR) which serve as a geographical marker for the posterior boundary of the lens epithelium. An example is provided in the inset, which is stained with Alexa Fluor488 phalloidin to label filamentous actin. The vertical dashed line is included to roughly depict the end of the transitional zone (TZ) and the beginning of the GZ. **B**: EdU-positive cells in the GZ of an 8-month old lens. **C**: An example of labeling of S-phase cells using an antibody to BrdU in an explant from a 1-month-old lens. Consistent identification of the germinative zone is complicated by distortion of the explant during dissection. During dissection, there is unavoidable damage to the lens epithelium (asterisks). Finally, the explant is difficult to keep flattened, leaving areas of the explant out of focus and less intense, making quantification more difficult. **D**: Lens explant from an 8-month-old mouse labeled with BrdU. **E**: Quantification of the percentage of lens cells in S-phase (EdU-positive) from an 8-month-old Balb/c mouse kept under normoxic conditions (room air; 21% O_2_) compared to a mouse breathing 60% O_2_. The asterisk indicates a p<0.05). Total cells (green) were stained with the vital dye, DRAQ-5. Scale bars: 10 μm (**A**, **B**) and 50 μm (**C**, **D**).

The advantages of the EdU/whole lens method are illustrated in Figure 2. EdU (Figure 2A,B) labels epithelial cells in S-phase to a level that is comparably to staining obtained with BrdU (Figure 2C,D). The number of S-phase-positive cells is much higher in the germinative zone of lenses from younger (1-month-old) mice (Figure 2A,C) compared to the germinative zones from older (8-month-old) mice (Figure 2B,D). The germinative zones visualized in whole lenses using EdU were undamaged, allowing for more thorough evaluation of S-phase indices (Figure 2A,B). In contrast, lens explants stained for BrdU contained regions of significant damage, making them difficult to analyze and harder to quantify (Figure 2C,D). It is also important that epithelial cells in explants are not always in focus, due to unavoidable folding of the lens capsule. Flattening of whole lenses allows for all epithelial cells in a microscope field to be consistently oriented and focused.

The inset in Figure 2 shows staining with phalloidin to label filamentous actin (F-actin). The meridional rows demarcate the onset of the posterior of the lens. They serve as a geographical marker for lens orientation and also allow for easy alignment of lenses along their equators. The images shown in Figure 2A,B were captured in the following manner: 1) Lenses were aligned with their meridional rows oriented vertically, parallel to the Y-plane, 2) once the meridional rows were located, the visual field for data capture was found by moving the stage of the confocal microscope (the visual field) horizontally (in the X-plane) toward the anterior pole of the lens until the meridional rows were excluded from the field by one cell width. These simple steps provided a consistent orientation of the transition and germinative zones for visualization and counting. The lenses can then be gently rotated along their equator, allowing for quantification of total and EdU-positive cells around the circumference of the lens.

We previously found that creating hyperoxic conditions in the eye by having rats or mice breath 60% O_2_ significantly increased lens epithelial cell proliferation in vivo [7,8]. These studies were performed on lens explants and used BrdU labeling to quantify the S-phase index. To confirm that quantifying the S-phase index in whole lenses provides an accurate assessment of lens cell proliferation, we performed similar studies using the EdU/whole lens method. Exposing 8-month-old Balb/c mice to 60% O_2_ overnight tripled the percentage of EdU-positive cells, compared to animals breathing room air (21% O_2_; Figure 2E), a result similar to that obtained with BrdU labeling [8].

### Double labeling with fluorescent-conjugated antibodies in adult whole lenses

**Figure 3:**
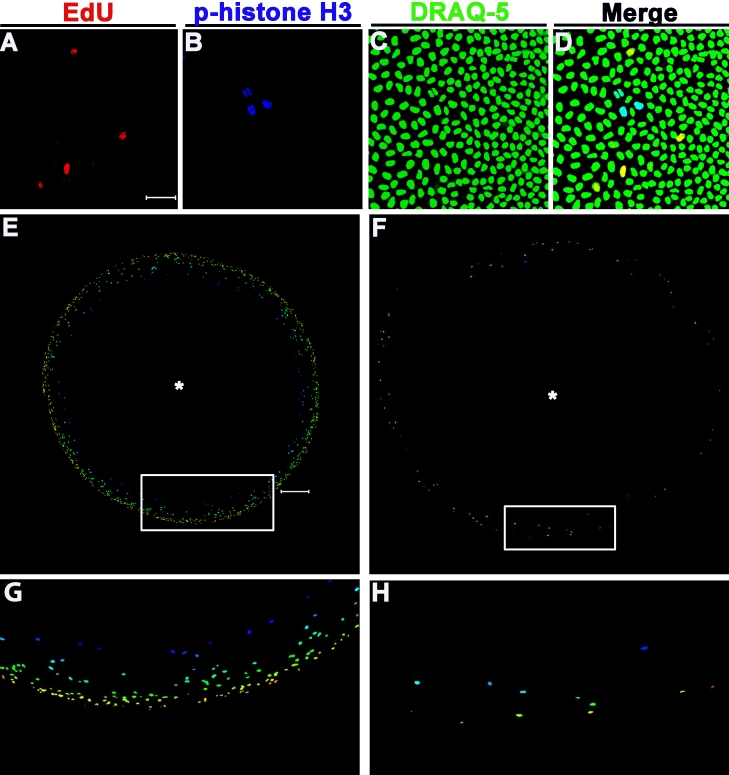
Cell cycle detection and the proliferative landscape of whole lenses. **A**: EdU-positive (S-phase) cells in the whole lens from an 8-month-old lens. **B**: Cells undergoing mitosis are labeled using a fluorescent-conjugated antibody to phosphorylated-histoneH3 in an 8-month old lens. **C**: Total cells in an image field labeled with DRAQ-5. **D**: Merged image of all three labels detecting two phases of the cell cycle in the germinative zone of a whole lens. **E**: Whole lenses were placed anterior side down. Image stacks (275 μm) were acquired in the Z-plane from the center of the anterior pole of the lens, demarcated by the asterisk. Z-stacks were then projected in the Y-plane and joined together to re-create the entire lens image. Depth coding was then applied to each stack to provide relevant distance from the initiation point of each z-stack. Cooler colors are closer to the asterisk (origin of the z-stack) than are warmer colors. Using this method, the germinative zone of an entire lens can be seen in a 1-month old lens. **F**: Reconstruction of an 8-month-old lens showing the decrease in the number of cells in S-phase with age. **G**: A larger image of the boxed area in (**E**), showing the color scheme of the depth coding application. **H**: Larger image of the boxed area in (**F)**. Scale bars: 10 μm (**A**-**D**) and 200 μm (**E**-**H**).

We performed immunostaining in adult lenses using a fluorescently-conjugated primary antibody against phosphorylated-histoneH3 (p-hH3), a marker for the mitotic phase of the cell cycle [9]. Double-staining of lenses with p-hH3 and EdU permits simultaneous observation of epithelial cells in the S- and M-phase of the cell cycle (Figure 3A-D). This approach should be applicable to other primary antibodies.

### Imaging whole lenses

Using confocal microscopy, we were able to reconstruct the entire germinative zone of a 1-month-ld lens (Figure 3E). To do this, the lens was oriented with the anterior pole down on a glass coverslip. From the center of the lens, 275 μm-thick stacks were imaged in the Z-plane. To reconstruct the lens, the z-stacks were flattened in a single plane (projected) and depth coded. Depth coding allows for the spatial reference of labeled nuclei. Those nuclei coded in cooler colors, such as blue or green, are closer to where the z-stack was initiated. Conversely, those labeled with warmer colors, such as orange, yellow, and red are farther away from where the z-stack was initiated. From the representative image shown, it is evident that EdU-positive nuclei are located more anteriorly in the epithelium in the 1-month-old lens, compared to the 8-month-old lens (Figure 3F). In both cases the majority of EdU-positive cells are found along the periphery of the image, in the germinative zone near the lens equator.

### Advantages of the whole-lens method

There are several advantages to imaging whole lenses compared to the standard procedure involving lens explants: 1) isolation of whole lenses from six or more animals can be accomplished in minutes, whereas dissecting explants from a similar number of lenses can take hours and damages the lens epithelium and capsule during preparation 2) fixation, permeabilization, and detection of EdU can be done in hours, whereas BrdU detection takes two to three days, 3) BrdU detection requires harsh denaturation of the sample, which can destroy epitopes for antibody detection and alter cell morphology, 4) imaging whole lenses allows for imaging of the entire lens equator, while equatorial cells are often damaged during explantation and 5) all reagents needed for EdU detection are small molecules, permitting them to readily penetrate the lens capsule, speeding staining and washing and reducing background staining. The EdU/whole lens protocol is compared to the standard BrdU/lens explant method in Table 1.

**Table 1:** Comparison of the steps required for BrdU and EdU labeling of lens explants or whole lenses.

**Step**	**BrdU/Explant**	**Time**	**EdU/Whole**	**Time**
1	Inject BrdU, wait for incorporation	1 h	Inject EdU, wait for incorporation	1 h
2	Isolation of lenses/preparation of explants	2–4 h	Isolation/cleaning lenses	30 min
3	Fixation	1 h	Fixation	10 min
4	Wash	30 min	Wash	10 min
5	HCl Denature	1 h	Permeabilization	1 h
6	Wash	1 h	Wash	10 min
7	Block	1–2 h	“Click” reaction/ DRAQ-5 staining	1 h
8	Primary Antibody	~12 h	Wash	1 h
9	Wash	1 h	Ready to View	
10	Secondary Antibody plus nuclear stain	2 h		
11	Wash	1 h		
12	Ready to view			
Total		~23–26 h (2–3 laboratory days)		~5 h (one laboratory day)

### Limitations of the whole-lens method

Although the method described has several advantages over conventional techniques for identifying S-phase cells in the lens, it does have limitations. The EdU method benefits from using only small molecules, which readily penetrate the lens capsule. However, double-staining whole lenses with EdU and antibodies can be difficult, requiring long incubation in the antibody and extensive washing to reduce background staining. The double-labeling for p-hH3, described above, benefitted from the use of a fluorescently-labeled primary antibody, rather than indirect immunofluorescence using a fluorescently-labeled secondary antibody. Difficulties in using standard immunofluorescence methods may limit the ability to co-localize antigens in adult lenses, making lens sections or explants the preferred method for some applications.

We also found that compressing the lens sometimes ruptured the lens capsule. This can be avoided by collecting Z-stacks of images of an uncompressed lens or a slightly compressed lens on a confocal microscope and then projecting the Z-stack into a single image.
